# Notes from the Field: *Vibrio cholerae* Serogroup O1, Serotype Inaba — Minnesota, August 2016

**DOI:** 10.15585/mmwr.mm6636a6

**Published:** 2017-09-15

**Authors:** Victoria Hall, Carlota Medus, George Wahl, Alida Sorenson, Melanie Orth, Monica Santovenia, Erin Burdette, Kirk Smith

**Affiliations:** ^1^Epidemic Intelligence Service, CDC; ^2^Minnesota Department of Health; ^3^Minnesota Department of Agriculture; ^4^Division of Foodborne, Waterborne, and Environmental Diseases, National Center for Emerging and Zoonotic Infectious Diseases, CDC.

On August 20, 2016, the Minnesota Department of Health (MDH) was notified of a case of *Vibrio cholerae* infection. The isolate was identified as serogroup O1, serotype Inaba at MDH. CDC determined that the isolate was nontoxigenic. The patient was a previously healthy woman, aged 43 years, with history of gastric bypass surgery. On August 16, she experienced profuse watery diarrhea, vomiting, abdominal cramps, and headache. On August 18, she sought care and submitted the stool specimen that yielded the *V. cholerae* isolate. She reported no recent travel. However, she had consumed ceviche made with raw shrimp and raw oysters at restaurant A on August 14, 49 hours before illness onset. Her husband had a similar illness with a similar incubation period after eating the same foods at restaurant A.

On August 22, MDH sanitarians visited restaurant A and obtained tags and invoices for oyster and shrimp products; the oysters were a product of the United States, and the shrimp was a product of India. Sanitarians also gathered patron contact information and credit card receipts for August 12–14. Two additional patrons reported experiencing a gastrointestinal illness that met the case definition of three or more episodes of watery stool in a 24-hour period within 5 days of eating at restaurant A; one reported eating ceviche and oysters at restaurant A. Review of complaints to the MDH foodborne illness hotline revealed a previous complaint from two persons who reported experiencing watery diarrhea after eating raw shrimp ceviche (but no oysters) at restaurant A on August 2. These persons did not provide stool specimens, but their gastrointestinal illnesses met the case definition, resulting in a total of six cases, including one laboratory-confirmed case. No other *V. cholerae* O1 Inaba cases were reported in the United States during this outbreak.

The Minnesota Department of Agriculture facilitated sampling of shrimp at the distributor from the same lots served at restaurant A on August 14, and most likely during August 2–13, and sent them to the Food and Drug Administration for culture. Shrimp samples yielded *V. cholerae* non-O1, non-O139, but *V. cholerae* O1 was not isolated. In response to the outbreak results, restaurant A placed consumer warnings on their menus about the risks of consuming raw or undercooked food items and identified raw menu items for consumers. Restaurant A also focused on other actions that might facilitate reduction of *V. cholerae*, including appropriate freezing of food items, and allowing raw food items to soak in lime juice before being served, rather than serving the items immediately after adding lime juice (*1*,*2*).

*V. cholera* has over 150 serogroups and has been identified in a wide range of aquatic life, including seafood (*3*). Whereas multiple serogroups can cause vibriosis, only serogroups O1 and O139 that also contain the cholera toxin are classified as causes of cholera (*4*). Previous studies have documented the presence of nontoxigenic *V. cholerae* O1 from environmental and shrimp samples in India and Southeast Asia (*5*–*7*).

This outbreak of domestically acquired, nontoxigenic *V. cholerae* infections, likely from shrimp consumption, included the first *V. cholerae* O1 case identified in a nontraveler in Minnesota since active surveillance for *Vibrio* began in 1996. Since 1996, MDH has detected 26 *V. cholerae* infections, 21 (81%) of which were non-O1, non- O139, and five of which were O1. Among the four O1 type cases identified before the current outbreak, all patients had a recent travel history to Micronesia or India. This outbreak demonstrates the importance of investigating all seafood eaten by patients with vibriosis. In addition, investigators should include nontoxigenic *V. cholerae* as a possible etiology of domestic foodborne outbreaks, particularly when foods eaten include those from *V. cholerae* O1–endemic areas.
